# Exploring Mental Health Professionals’ Perspectives of Text-Based Online Counseling Effectiveness With Young People: Mixed Methods Pilot Study

**DOI:** 10.2196/15564

**Published:** 2020-01-29

**Authors:** Pablo Navarro, Jeanie Sheffield, Sisira Edirippulige, Matthew Bambling

**Affiliations:** 1 Kids Helpline yourtown Brisbane Australia; 2 School of Psychology University of Queensland Brisbane Australia; 3 Centre for Online Health University of Queensland St Lucia Brisbane Australia; 4 Central School of Medicine Royal Brisbane and Women's Hospital The University of Queensland Brisbane Australia

**Keywords:** mental health, child health, adolescent health, distance counseling, mHealth, applied psychology, psychological processes

## Abstract

**Background:**

Population-based studies show that the risk of mental ill health is highest among young people aged 10 to 24 years, who are also the least likely to seek professional treatment because of a number of barriers. Electronic mental (e-mental) health services have been advocated as a method for decreasing these barriers for young people, among which text-based online counseling (TBOC) is a primary intervention used at many youth-oriented services. Although TBOC has shown promising results, its outcome variance is greater in comparison with other electronic interventions and adult user groups.

**Objective:**

This pilot study aimed to explore and confirm e-mental health professional’s perspectives about various domains and themes related to young service users’ (YSUs) motivations for accessing TBOC services and factors related to higher and lower effectiveness on these modalities.

**Methods:**

Participants were 9 e-mental health professionals who were interviewed individually and in focus groups using a semistructured interview. Thematic analysis of qualitative themes from interview transcripts was examined across the areas of YSU motivations for access and factors that increase and decrease TBOC effectiveness.

**Results:**

A total of 4 domains and various subthemes were confirmed and identified to be related to YSUs’ characteristics, motivations for accessing TBOC, and moderators of service effectiveness: user characteristics (ie, prior negative help-seeking experience, mental health syndrome, limited social support, and perceived social difficulties), selection factors (ie, safety, avoidance motivation, accessibility, and expectation), and factors perceived to increase effectiveness (ie, general therapeutic benefits, positive service-modality factors, and persisting with counseling despite substantial benefit) and decrease effectiveness (ie, negative service-modality factors).

**Conclusions:**

Participants perceived YSUs to have polarized expectations of TBOC effectiveness and be motivated by service accessibility and safety, in response to several help-seeking concerns. Factors increasing TBOC effectiveness were using text-based communication, the online counselor’s interpersonal skills and use of self-management and crisis-support strategies, and working with less complex presenting problems or facilitating access to more intensive support. Factors decreasing TBOC effectiveness were working with more complex problems owing to challenges with assessment, the slow pace of text communication, lack of nonverbal conversational cues, and environmental and connectivity issues. Other factors were using ineffective techniques (eg, poor goal setting, focusing, and postcounseling direction) that produced only short-term outcomes, poor timeliness in responding to service requests, rupture in rapport from managing service boundaries, and low YSU readiness and motivation.

## Introduction

### Background

Over the past 2 decades, electronic mental (e-mental) health services have emerged as a promising avenue for intervention because of the advantages they have in accessibility, efficiency, and effectiveness over traditional service provision [1-7]. In particular, these services have been advocated for use in public health strategies for young people aged 10 to 24 years [6-10], who have the highest risk for developing emotional and mental health problems and are the least likely to seek professional treatment because of a number of help-seeking obstacles [11-18].

Online counseling is a common e-mental health intervention offered at youth helplines across the world [7,19] and is described by Barak and Grohol [20] as a “*mental health intervention between a patient [or a group of patients] and a therapist, using technology as the modality of communication*” (p. 157). Herein, text-based online counseling (TBOC) is a common typology that allows asynchronous (eg, email and forums) or synchronous (eg, Web chat) communication between young service users (YSUs) and online counselors. Although face-to-face counseling remains the preferred counseling modality for most YSUs [21-23], a growing number of YSUs are expressing a preference for online interventions [21,22].

Studies show that the most common factors influencing a YSU’s selection of TBOC services are the heightened levels of safety associated with interface-related privacy, anonymity, control, and reduced nonverbal feedback from the online counselor; increased accessibility resulting from convenience, affordability, and ease of access; and polarized minimal-to-heightened expectations about the outcome of counseling sessions [24-32]. YSUs are observed to access TBOC services most commonly for support related to mental health, relationships, and information-related requests (eg, medicolegal, service, and resource queries), expressing a higher degree of problem burden and distress relative to those who access nontext-based counseling modalities [7,33,34]. Although promising evidence for the effectiveness of TBOC interventions is starting to emerge [35-39], greater variance has been observed in the impact of these interventions for YSUs [33,40-42] than adults [43-48].

This raises questions about the factors that increase and decrease the effectiveness of these modalities, which may contribute to these dissimilar findings. We are especially interested in the perspective of e-mental health professionals who research, develop, and provide these services for whom there is little research to examine their experiences. Existing literature suggests that online counselors generally endorse the effectiveness of TBOC for YSUs, describing the tandem benefit of reduced emotional proximity to the online counselor and subsequently increased perceptions of safety that promote uninhibited discussion [49,50]. However, online counselors express concern that presenting problems featuring severity or risk may be less suitable for text-based modalities because of difficulties with comprehensive assessment; the slow pace of typed communication and not being able to offer full interventions within session time limits; misunderstandings from an absence of nonverbal cues or the presence of literacy issues, connectivity issues, and YSU distractibility; and avoidance of counseling content [49-53].

In terms of the counseling approaches used at TBOC services, emerging research indicates that many online counselors have a preference for supportive counseling frameworks with solution-focused and cognitive behavioral interventions (eg, psychoeducation, action planning, and referral) [25,33,34,41,50,52,54]. Yet, research also indicates that online counselors typically spend more time building rapport and assessing a YSU’s presenting problem than setting goals, working toward them, or offering complex interventions beyond information giving or service referral [41,52,55,56]. The reasons for this preference are unclear. Some research suggests that this may be attributable to enduring challenges with comprehensive clinical assessment and the slower pace of TBOC modalities [41,52,55,56]. At least one study has revealed that engaging in more sessions was associated with greater counseling depth and alleviation in YSU psychological distress, with problem clarification and action planning processes being most correlated with reductions in distress in YSUs [41].

In view of the aforementioned literature, online counselors working via TBOC modalities appear to generally affirm its benefits for YSUs. At the same time, they identify issues related to working with more complex presentations because of limitations with communicating in a text-based milieu, which may explain the greater outcome variance YSUs experience on TBOC modalities.

### Objective

This pilot study sought the perspective of e-mental health professionals in confirming the following domains and themes indicated by the literature about YSU’s TBOC experiences for measurement in a larger future study: (1) motivations for selecting TBOC services (eg, safety, access, and expectations), therapeutic benefits experienced during sessions (eg, catharsis and validation), (3) experiences of TBOC ineffectiveness (eg, not making any progress), and (4) reasons for persisting with TBOC services when perceived to be ineffective.

The secondary aim of the study was to identify further constructs of interest for investigation in our future study by asking e-mental health professionals to expand on any endorsed domains. It was expected that e-mental health professionals would endorse each domain being measured, consistent with the available literature. This study is unique in its specific examination of e-mental health professional’s perspectives of TBOC effectiveness and ineffectiveness factors, given their role in researching, developing, and facilitating text-based interventions. It is hoped that these findings will provide more a nuanced theory-practice perspective about the factors that may contribute to TBOC effectiveness variance for YSUs.

## Methods

### Design

The study used qualitative individual and focus group interviews, which were determined to be the most appropriate method of eliciting participants’ perceptions about YSUs’ motivations for TBOC service use and factors moderating effectiveness. Focus group interviews are described as an effective method of generating discussion about a subject area that enables participants to compare attitudes, values, and understanding in a given subject area [57]. Participants who were unable to attend focus groups were offered individual interviews as an alternative.

The semistructured interview used was developed for this study and informed by existing TBOC literature. It sought to confirm domains and themes identified in our objectives statement with participants and identify new domains and themes using 3 common steps: (1) the presentation of information about a target TBOC domain, (2) asking participants to indicate agreement or disagreement with having observed the target domain directly (during clinical consultations) or indirectly (during the course of academic research) with YSUs, and (3) inviting participants to elaborate on their experiences with the domain or its related themes and subthemes.

The interview guide used for both individual and focus group interviews can be viewed in Multimedia Appendix 1. The Consolidated criteria for REporting Qualitative research checklist was also completed for this study and can be found in Multimedia Appendix 2.

### Participants

A total of 10 participants were approached and recruited for the study. Exclusion criteria in the study were having less than 5 years’ experience working in e-mental health services in the capacity of clinician or researcher and having less than 5 years’ experience working with YSUs. Of 10 participants, 1 was excluded from the study because of interview bias resulting from their having substantially limited knowledge and experience about TBOC services to make a meaningful contribution to the study.

The final sample of participants was 9 e-mental health professionals (5 females and 4 males), aged 27 to 67 years (mean 42.6 years, SD 14.3), currently working in direct client services (6/9) and research (2/9) and tribunal (1/9) roles; and employed in community mental health (6/9), tertiary education (2/9), and government (1/9) settings. Of the 9 participants, 6 had collegial relationships with the interviewer and were aware of the broad area of research being conducted (TBOC) but did not know specifics of the study.

Participants possessed tertiary qualifications in mental health (undergraduate degree=3/9 postgraduate degree=3/9 and doctoral degree=3/9), and they described their professional roles to be psychologists, social workers, counselors, and health scientists. Participants had 8.5 years’ average experience working in e-mental health settings and 8.1 years’ average experience working with YSUs in mental health settings.

### Procedure

Ethical approval for the study was obtained from the University of Queensland’s Behavioral & Social Sciences Ethical Review Committee. The primary author was the interviewer of this study. He was acting in the role of counseling psychologist for young people and adults at the time of the study and has postgraduate training and experience in conducting research interviews.

Participants were recruited using a word-of-mouth sampling technique, owing to the need to select e-mental health experts with diverse understanding of youth mental health and TBOC interventions. All interviews were conducted solely with the interviewer and participants. Overall, 4 participants attended individual interviews at university and hospital workplaces, and 6 participants attended 3-person mini focus group interviews at Kids Helpline. Mini focus group interviews were conducted with Kids Helpline staff because of the challenges in arranging for participants to attend traditionally sized focus, given its shift work environment. Focus groups and interviews were approximately 60 min duration in a face-to-face format (1 participant attended a telephone interview). All interviews were audio recorded and transposed to text by a professional transcription service, with the names of participants changed to ensure anonymity. All participants were followed up via email after their interviews were transcribed to ask them clarifying questions about their responses, where information was unclear in the interviewer’s notes or audio transcripts.

### Data Analysis

Thematic analysis was the approach taken to understanding participant’s perceptions about the characteristics and motivations of YSUs accessing TBOC and factors moderating the effectiveness of these modalities. The thematic analysis methodology described by Braun and Clarke [58] was used, involving the process of developing familiarity with data, generating initial codes, searching for themes, reviewing themes, defining and naming themes, and producing a report of findings.

The primary author initially read all transcripts, and then 1 interview transcript was assigned to each of 5 independent analysts. Analysts manually defined a set of preliminary codes that did not necessarily conform to the language used in the interview guide. The primary author then met with each analyst independently to compare their codes and search for themes in the data. Coding discrepancies between the primary author and independent analyst were discussed until a consensus was obtained. Qualitative themes were only rated once for each participant describing them, irrespective of the number of times that theme was identified. The primary author then reviewed themes across all datasets to search for overlapping themes generated by analysts and collapsed these into the fewest themes possible and renamed them accordingly. The final stages of analysis involved the authors reviewing themes and subthemes to ensure that coded extracts were valid, logical, and reasoned.

A frequency count was applied to themes to identify their commonality for our future planned studies, wherein we seek to further examine those with higher occurrence. Ratings of *very strong*, *strong, moderate,* and *weak* were given if >80%, >50%, >30%, and >10% of participants endorsed the theme, respectively, with cutoffs influenced by the effect size literature. Given the small sample size in this pilot study, themes endorsed by fewer than 2 participants were excluded from the analysis on the grounds of low generality.

## Results

### Overview

**Figure 1:**
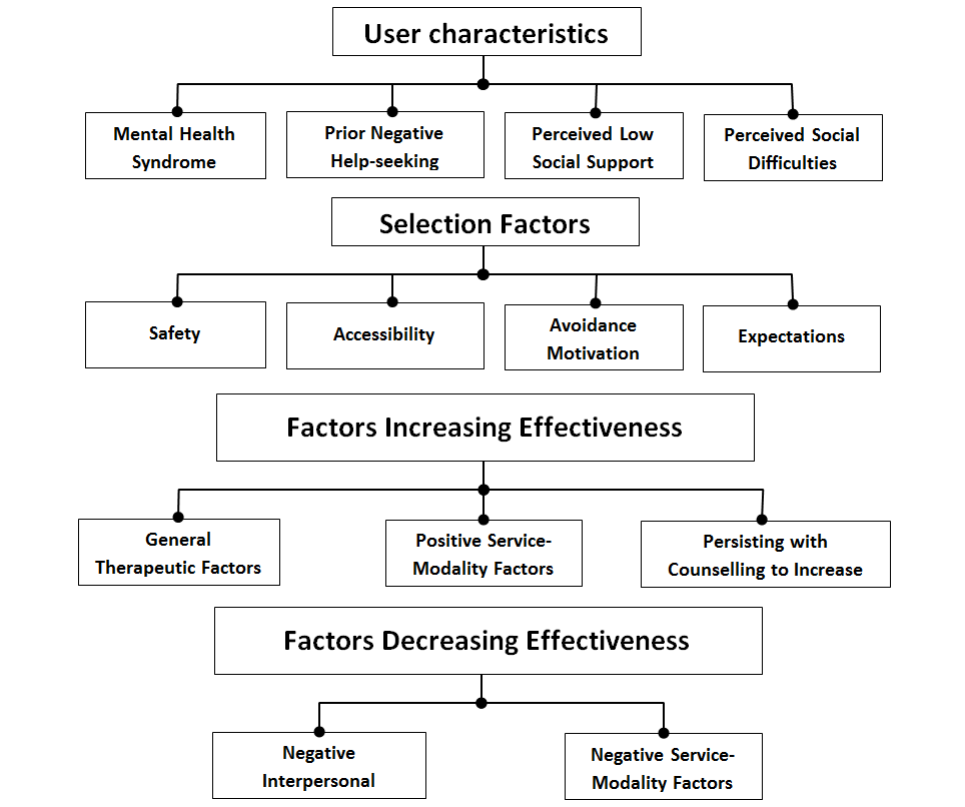
Overview of primary domains and themes identified by text-based online counseling providers.

Participants confirmed 3 domains, and identified a unique domain (ie, *user characteristics*), that were related to YSUs’ motivations for selecting TBOC services and their effectiveness. The following sections present qualitative data describing the experiences of participants in these 4 areas. Figure 1 shows an illustrative overview of primary domains and themes identified in the study.

### User Characteristics

*User characteristics* emerged as a unique domain for which participants described factors related to context, experience, and perception believed to precede a YSU’s selection of TBOC modalities. Participants described 4 themes related to *user characteristics: prior negative help-seeking experience, mental health syndromes, limited social support*, and *perceived social difficulties*. An overview of domains and themes related to *user characteristics* is presented in Table 1.

The view that many YSUs select to use TBOC services because of a *prior negative help-seeking experience* was described by 5 of 9 participants. Participant responses were similar. The key aspect described was that YSUs often sought the safety of TBOC services following negative responses from family to their help-seeking efforts or rupture in rapport with professional workers.

**Table 1:** Overview of themes identified by text-based online counseling providers about perceived user characteristic domains.

Domains and themes (user characteristics)	Strength of theme	Examples of theme
Prior negative help-seeking experience	Strong	*Sometimes it occurs... there may have been a rupture within the therapeutic relationship with a face to face worker, so [YSUs] may retreat to an online service because it's perceived to be more comfortable for them that way.*
Mental health syndrome	Strong	*If you have a look at the research that Kids Helpline has done and look at the frequency of problems coming up in the online environment... what you’ll find is that you get a higher frequency of mental health problems and suicidality and other indicators of more severe disturbance in the online environment suggesting that people who are really quite distressed and maybe somewhat paranoid have a strong preference to that environment compared with the telephone environment.*
Limited social support	Moderate	*When you start to explore who else [YSUs can] talk to, they feel like there is no one else that they can talk to about a particular issue. That's the reason why they are contacting in this way.*
Perceived social difficulties	Moderate	*You'll hear [YSUs] reflect on having an introverted personality, and that they'll... they just find that they are able to express themselves better in this setting.*

The view that many YSUs present to TBOC services with *mental health syndromes* was described by 5 of 9 participants. Participant responses were similar. The key aspect described was that TBOC services were commonly used by YSUs with mental ill health because of the safety associated with communicating in text-based online environments.

The view that many YSUs experience *limited social support* was described by 3 of 9 participants. Participant responses were variable. Key aspects that emerged were about YSUs seeking out TBOC services to compensate for a low-density support network, perceived inability to talk to people in their existing support network, and the desire to add more support people to their established support network.

The view that many YSUs experience *perceived social difficulties* was described by 3 of 9 participants. Participant responses were variable. Key aspects that emerged were that some YSUs perceiving themselves to have communication difficulties, introverted personalities, or undesirable physical characteristics that make them socially anxious.

### Selection Factors

Participants confirmed 4 themes related to their perceptions of YSUs’ motivations for selecting TBOC modalities for counseling support: *safety, accessibility, avoidance motivation*, and *expectations*. An overview of domains and themes related to *selection factors* is presented in Multimedia Appendix 3.

#### Safety

All participants shared perceptions that confirmed *safety* as the most important motivator underpinning a YSU’s selection of TBOC modalities. A total of 3 subthemes were identified within this theme.

The view that TBOC felt safer to YSUs because of *increased privacy* from its text-based interface was described by 8 of 9 participants. Participant responses were similar. The key aspect described was that YSUs were motivated to conceal their help-seeking efforts from others in their immediate environment, mostly their parents.

The view that TBOC felt safer to YSUs because of *increased control and autonomy* over the counseling process was described by 7 of 9 participants. Participant responses were variable. Key aspects that emerged were about YSUs being wanting control over making disclosures and when counseling sessions end and independence in accessing a mental health service, especially when parental gatekeeping was a barrier to care.

The view that TBOC felt safer to YSUs because of *increased anonymity* when using text-based interfaces was described by 7 of 9 participants. Participant responses were variable. Key aspects that emerged were about YSUs feeling able to discuss more sensitive content when online counselors could not see them or did not personally know them and conceal information leading to their personal identification in the community, especially when disclosing risk information.

#### Accessibility

All participants shared perceptions that confirmed *accessibility* to be an important motivator underpinning YSUs’ selection of TBOC modalities. A total of 8 subthemes were identified within this theme.

The view that the *convenience and flexibility* of TBOC services increased YSU access to care was described by 8 of 9 participants. *Convenience* and *flexibility* determined to be interrelated by analysts because of their frequent cooccurrence and similarities in their features. Participant responses were variable. Key aspects that emerged were about the convenience of accessing TBOC services from one’s own device and the flexibility of accessing TBOC services from any location or at any time of day.

The view that TBOC services increased YSU access to care by allowing *faster access to counseling* was described by 8 of 9 participants. Participant responses were variable. Key aspects that emerged were about YSUs being able to access support at the moment of distress or crisis rather than *circumnavigating waiting lists* (see subtheme) and the disadvantage of accessing TBOC services when in crisis or distress when delays were created by high service demand.

The view that the *affordability* of TBOC services increased YSU access to care was described by 6 of 9 participants. Participant responses were similar. The key aspect described was about the unaffordability of counseling sessions to YSUs, given their developmentally limited access to money.

The view that *sourcing one’s own support* by using TBOC services increased YSUs access to care was described by 6 of 9 participants. Participant responses were variable. Key aspects that emerged were about YSUs being able to use TBOC services to overcome restricted access to devices or internet access, systemic barriers to accessing mental health care, accessibility issues related to service location and appointment availability, and privacy-related issues to service use.

The view that TBOC services increased YSUs’ access to care by *circumnavigating waiting lists* was described by 5 of 9 participants. Participant responses were similar. The key aspect described was about YSUs being able to access care quickly because of the absence of waiting lists and a shorter intake assessment process, relative to face-to-face services.

The view that TBOC services increase YSU access to *counseling in areas of low service density* was described by 3 of 9 participants. Participant responses were similar. The key aspect described was about YSUs being able to access counseling when living in areas with no services available or services only available at very far-to-reach distances.

The view that text-based communication was a *preferred way* (for YSUs) *to communicate* that increases their likelihood of accessing care was described by 2 of 9 participants. Participant responses were similar. The key aspect described was that availability of internet-ready devices, and the popularity of text-based communication within youth culture makes TBOC services more accessible and, therefore, appealing to YSUs.

The view that TBOC services help with *overcoming transportation issues* to care was described by 2 of 9 participants. Participant responses were similar. The key aspect described was that TBOC services increased service access because they do not require YSUs to have a license, vehicle, or access to transportation.

#### Avoidance Motivation

*Avoidance motivation* emerged as a unique theme that was perceived by all participants to underlie YSUs’ perceptions of *safety* about TBOC services. A total of 6 subthemes were identified within this theme.

The view that YSUs select TBOC services to minimize the chance of *being overheard or seen attending a service* was described by 8 of 9 participants. Participant responses were similar. The key aspect described was YSUs had concerns about the social consequences of being discovered accessing counseling by one’s family or peers.

The view that YSUs selected TBOC services to *minimize difficult emotions* during counseling sessions was described by 7 of 9 participants. Participant responses were similar. The key aspect described was that YSUs feel uncomfortable with becoming embarrassed, vulnerable, or overwhelmed during counseling sessions, which were perceived to be minimized when using TBOC rather than face-to-face modalities.

The view that YSUs selected TBOC services to minimize *privacy and security concerns* was described by 6 of 9 participants. Participant responses were variable. Key aspects that emerged were about YSUs feeling concerned about who might be able to access their private information, whether online counselors might share their personal information with known others, and their need to withhold or alter personal identifying information to enhance the security of their data.

The view that YSUs selected TBOC services to minimize the *counselors’ reaction* was described by 5 of 9 participants. Participant responses were similar. The key aspect described was about YSUs having concerns that online counselors might be unfriendly, judgmental, or needing to take undesired action based on content disclosed in sessions, motivating them to reduce interpersonal feedback cues.

The view that YSUs selected TBOC services to avoid *challenging conversations* was described by 5 of 9 participants. Participant responses were similar. The key aspect described was about YSUs feeling concerned that online counselors’ questions were uncomfortable, confronting, or may result in undesired action being taken based on disclosed content, motivating them to avoid these questions or lines of conversation.

The view that YSUs selected TBOC services to avoid *social interaction* was described by 3 of 9 participants. Participant responses were variable. Key aspects that emerged were about YSUs seeking to avoid social interaction owing to concerns about *perceived social difficulties* (see subtheme) and the time pressure to respond to an online counselor’s questions in a face-to-face environment.

#### Expectations

Most participants (7/9) shared perceptions that confirmed how YSUs’ *expectations* about TBOC outcome motivated their access to these services. A total of 4 subthemes were identified within this theme.

The view that YSUs had *few expectations* of TBOC services was described by 5 of 9 participants. Participant responses were variable. Key aspects that emerged were about YSUs either having no expectations of TBOC services or being encouraged by loved ones to use these services rather than having personal investment in the outcome.

The view that YSUs had *high treatment expectations* of TBOC services was described by 4 of 9 participants. Participant responses were variable. Key aspects that emerged were about YSUs expecting that TBOC services could assist them with issues of higher complexity and/or in a faster than realistic manner.

The view that a *prior successful interaction* on TBOC services was related to elevated YSU expectations was described by 4 of 9 participants. Participant responses were similar. The key aspect described was that YSUs were more likely to access TBOC services in return if they had previously had a beneficial counseling session.

The view that YSUs saw TBOC services as *equivalent to other counseling modalities* was described by 3 of 9 participants. Participant responses were similar. The key aspect described was that YSUs tended to view all modalities of counseling as being similar in process and producing similar outcomes.

### Factors Perceived to Increase Effectiveness

Participants confirmed 3 themes to be related to factors they perceived to increase the effectiveness of TBOC: *general therapeutic benefits, positive service-modality factors*, and *persisting with counseling to increase benefit*. An overview of domains and themes related to *factors perceived to increase effectiveness* is presented in Multimedia Appendix 4.

#### General Therapeutic Benefits

This theme was confirmed by all participants who perceived that the interpersonal skills and experiences universal to counseling were related to TBOC effectiveness. A total of 3 subthemes were identified within this theme.

*Feeling listened to and understood* was described to be effective by 3 of 9 participants. Feeling *listened to* and *understood* were distinct subthemes that were combined by analysts because of their frequent cooccurrence and similarities in features. Participant responses were similar. The key aspect described was that YSUs feeling listened to and understood by online counselors was related to TBOC effectiveness.

*Catharsis or debriefing* was described to be effective by 2 of 9 participants. Participant responses were similar. The key aspect described was that YSUs sharing or processing their experiences with online counselors as a form of minimal intervention was related to TBOC effectiveness.

*Feeling normalized and validated* was described by 2 of 9 participants. Participant responses were similar. Feeling *normalized* and *validated* were distinct themes that were combined by analysts because of their frequent cooccurrence and similarities in features. The key aspect described was that YSUs feeling like their presenting problems were normal and accepted by the online counselor was related to TBOC effectiveness.

#### Positive Service-Modality Factors

This theme was confirmed by all participants who discussed service and modality factors perceived to be associated with increased TBOC effectiveness. A total of 7 subthemes were identified within this theme.

*Working with less complex presenting problems* was described by 6 of 9 participants to be related to increased TBOC effectiveness. Participant responses were similar. The key aspect described was that TBOC services were more effective when focused on issues with less complexity and pervasiveness that could benefit from short-term work.

Using TBOC services as a *stepping stone to more intensive counseling support* was described by 5 of 9 participants to be related to increased TBOC effectiveness. Participant responses were variable. Key aspects that emerged were that TBOC services were used by YSUs to trial and build comfort with the counseling process and as a pathway to face-to-face counseling, especially for those with complex presenting problems.

Of 9 participants, 4 described that text-based communication allowed YSUs a greater *ease of expression or thought organization*, which was related to increased TBOC effectiveness. Participant responses were variable. Key aspects that emerged were that text-based communication allowed YSUs to better articulate their internal experiences and leverage the latency of text-based communication to process their responses to counseling questions.

Another 4 of 9 participants discussed that YSUs valued TBOC modalities for their *crisis use*. Participant responses were similar. The key aspect described was that YSUs benefit from accessing TBOC services during a crisis because of the *faster access to counseling* (see subtheme) possible.

The *written record of information* produced by many TBOC modalities was described to be related to its effectiveness by 3 of 9 participants. Participant responses were similar. The key aspect described was that YSUs could benefit from accessing their counseling transcript to remember information and strategies provided during sessions.

Teaching YSUs *problem self-management strategies* was described to increase TBOC effectiveness by 2 of 9 participants. Participant responses were similar. The key aspect described was that interventions that facilitate self-control and self-management were associated with increased TBOC effectiveness.

*Therapist assistance* was described to increase TBOC effectiveness by 2 of 9 participants. Participant responses were similar. The key aspect described was that interventions were generally more effective when delivered by an online counselor as opposed to being delivered via self-help programs.

#### Persisting With Counseling to Increase Benefit

This theme was confirmed by 4 of 9 participants who described the perception that some YSUs did not appear to make progress toward their presenting problem when using TBOC services but continued to access irrespectively. Participant responses were variable. Key aspects that emerged were about YSUs accessing TBOC services without consideration of its effectiveness, being unable to articulate how TBOC services help them, and being motivated by the interpersonal benefits of the interaction rather than progress toward their presenting problem.

### Factors Perceived to Decrease Effectiveness

Participants confirmed 2 themes related to their perceptions about what decreased TBOC effectiveness: *interpersonal factors reducing effectiveness* and *negative service-modality factors*. An overview of domains and themes related to *factors perceived to decrease effectiveness* is presented in Multimedia Appendix 5.

#### Interpersonal Factors Reducing Effectiveness

This theme was confirmed among all participants who discussed interpersonal factors perceived to affect the TBOC process and reduce its effectiveness. A total of 11 subthemes were identified within this theme.

Of 9 participants, 7 described that a YSU’s *problem not improving or* (online counselors using) *ineffective techniques* reduced TBOC effectiveness. Participant responses were variable. Key aspects that emerged were about online counselors experiencing focusing and goal setting difficulties during sessions, a poor fit between the YSUs presenting problem and what TBOC modalities can provide, and a YSU’s expectations of TBOC not being met.

*Rupture in rapport* was described by 7 of 9 participants to reduce TBOC effectiveness. Participant responses were variable. Key aspects that emerged were about YSUs having negative interpersonal experiences with online counselors (eg, judgment or trivializing their problem), discomfort in counseling sessions that affected the therapeutic relationship, or feeling betrayed by the service when an external response was required in response to high-risk situations (eg, suicide attempt).

A YSU’s *focus on rapport instead of* (working toward) *goals* was described by 6 of 9 participants to reduce TBOC effectiveness. Participant responses were variable. Key aspects that emerged were about a minority of long-term YSUs developing a dependent relationship with TBOC services over time and how YSUs’ motivation to achieve their goals could shift to seeking *general therapeutic benefits* (see subthemes) when progress was limited.

*Poor conversion of counseling into postsession action* was described by 5 of 9 participants to reduce TBOC effectiveness. Participant responses were similar. The key aspect described was that YSUs were sometimes unwilling to use counseling content or unsure about which next steps to take to make progress toward overcoming their presenting problems.

YSUs *desiring greater service control* was described by 5 of 9 participants to reduce TBOC effectiveness. Participant responses were variable. Key aspects that emerged were about YSUs engaging in various behaviors to overcome TBOC service access boundaries, such as falsifying personal information, accessing through different service modalities, becoming more demanding of an online counselor’s availability, and disclosing crisis information to have their service requests prioritized.

*Incongruent client-counselor goals* were described by 4 of 9 participants to reduce TBOC effectiveness. Participant responses were variable. Key aspects that emerged were about disagreements between client-counselor regarding counseling goals and methods, such as whether to focus on supportive counseling versus clinical interventions, contacting a YSU’s mental health workers to create consistency in counseling work, and transitioning to other counseling modalities or services considered to be more suitable in addressing a YSU’s presenting problems.

Having *unclear goals or reasons for counseling* was described by 4 of 9 participants to reduce TBOC effectiveness. Participant responses were variable. Key aspects that emerged were about YSUs having low purpose, readiness, or motivation for accessing TBOC services; having difficulties articulating counseling goals or understanding what counseling can offer them; and interpersonal rather than clinical motivations for accessing TBOC services.

Experiencing *misunderstandings or literacy issues* were discussed by 4 of 9 participants to reduce TBOC effectiveness. *Misunderstandings* and *literacy issues* were combined by coders because of their frequent cooccurrence and having similar features. Participant responses were similar. The key aspect described was that TBOC services may not be suitable for YSUs with literacy, learning, or intellectual issues that may impair their ability to effectively use or comprehend text-based communication.

YSUs experiencing *low readiness, motivation, and self-confidence* for change was described by 4 of 9 participants to reduce TBOC effectiveness. Participant responses were similar. The key aspect described was that YSUs having the aforementioned experiences may not be in a state to make optimal use of counseling interventions.

The online *counselor going off topic or being unsure how to help* was described by 3 of 9 participants to reduce TBOC effectiveness. Participant responses were variable. Key aspects that emerged were that online counselors sometimes feel unclear about how to work with YSUs because of the absence of personal or assessment information provided and feeling less competent with online interventions.

Producing *outcomes with only short-term benefit* was described by 2 of 9 participants to reduce TBOC effectiveness. Participant responses were similar. The key aspect described was that short-term outcomes were sometimes indicative of less impactful *general therapeutic benefits* (see subtheme) rather than long-term impact on a presenting problem.

#### Negative Service-Modality Factors

This theme was confirmed among all participants who discussed service delivery and modality factors perceived to reduce TBOC effectiveness. A total of 8 subthemes were identified within this theme.

*Working with complex problems* was described by 8 of 9 participants to reduce TBOC effectiveness. Participant responses were variable. Key aspects that emerged were about complex problems having a poorer fit with TBOC modalities because of their *slow pace or lack of time to make a difference* and *challenges assessing presentations* (see subthemes); being less responsive to TBOC interventions in terms of directly addressing extrinsic factors, such as relationships or systemic issues; and requiring more specialized interventions and services to overcome.

A *poor timeliness of response* to service requests was described by 7 of 9 participants to reduce TBOC effectiveness. Participant responses were variable. Key aspects that emerged were that YSUs were less likely to use or reuse TBOC services if they had to wait too long because of the window being missed for support, change occurring with the passage of time, or their feeling upset and no longer wanting support.

*Challenges assessing presentations* were described by 6 of 9 participants to reduce TBOC effectiveness. Participant responses were similar. The key aspect described was that clinical assessment that took place entirely using text-based communication took longer and reduced its accuracy and comprehensiveness, especially when presenting problems were vague, complex, or involved risk-related issues.

TBOC services offering *too much anonymity or few barriers* was described by 5 of 9 participants to reduce their effectiveness. Participant responses were similar. The key aspect described was that reducing service barriers to entry sometimes resulted in YSUs accessing with unnecessary or inappropriate presentations.

The *lack of nonverbal conversational cues* present in text-based communication was described by 4 of 9 participants to reduce TBOC effectiveness. Participant responses were variable. Key aspects that emerged were that the absence of nonverbal conversational cues reduced identifiability of YSUs accessing with complex, crisis, or inappropriate presenting problems; feedback from YSUs about intervention effectiveness; and the effectiveness of rapport building and communication fluency.

*Environmental distractions* were described by 3 of 9 participants to reduce TBOC effectiveness. Participant responses were variable. Key aspects that emerged were that *environmental distractions* created delays in YSU responses and distractions away from counseling content and other personal activities (eg, schoolwork and vocational work).

The *slow pace or lack of time to make a difference* in TBOC sessions was described by 3 of 9 participants to reduce TBOC effectiveness. Participant responses were similar. The key aspect described was that text-based communication was slower than verbal communication, which reduced the amount of time available in a counseling session and the subsequent time to make a difference to one’s presenting problems.

*Technical or connectivity issues* were discussed by 2 of 9 participants to reduce TBOC effectiveness. Participant responses were variable. Key aspects that emerged were that when YSUs had a poor internet connection, there was a greater likelihood of session lag and dropout that resulted in session disruption and negative user experiences.

## Discussion

### Principal Findings

This study aimed to examine e-mental health professionals’ perspectives about what motivates YSUs to select TBOC services and the factors that influence the effectiveness of these interventions, with a view to confirm domains and themes described in the literature and identify novel phenomenon.

*User characteristics* was a unique domain in the study, encompassing participants’ views about YSUs’ contextual experiences that preceded contact with TBOC services. Participants’ view that YSUs commonly experience *mental health syndromes* is in line with literature indicating greater frequency and severity of mental health presentations on TBOC compared with other e-mental health modalities [7,19,25,33,59]. The view that YSUs often have *limited social support* is also consistent with the help negation literature and how young people have a tendency to negatively appraise and turn down available support, with the exception of helplines that are perceived to be a safer form of support [60]. Participants’ perspective that many YSUs have *prior negative help-seeking experiences* is in line with evidence that negative past help-seeking experiences are a substantial barrier to future help-seeking for young people [60], suggesting that they may turn to TBOC services following these responses to their help-seeking efforts. Finally, participants’ view that YSUs commonly experience *perceived social difficulties* was a unique finding indicating that in addition to communication preferences, some may prefer to access TBOC services because of social anxiety symptoms that are reduced when leveraging the safety of text-based communication.

Participants’ views confirmed *selection factors* as a domain describing the reasons YSUs seek TBOC services. *Safety* and *accessibility* were viewed as the strongest motivators for YSUs to access TBOC services consistent with existing literature about their desire for privacy, anonymity, and control [6,26,28,30,61] as well as their struggles with financial, gatekeeper, transportation, and convenience-related barriers to mental health care [23,30,62-65]. *Avoidance motivation* and its subthemes were a unique theme among participants, which provided context to YSUs’ underlying motivations for seeking *safety* from TBOC services as method of coping with threatening or uncomfortable situations. Finally, *expectations* of counseling were commonly viewed to have a polarized influence on YSUs access to TBOC services, consistent with existing literature [31,40].

Participants’ views confirmed *factors perceived to increase effectiveness* as a domain describing positive predictors of TBOC outcome. Herein, *general therapeutic benefits* stemming from online counselors’ interpersonal skills were strongly perceived to be related to TBOC effectiveness, in line with literature indicating the commonality and effectiveness of person-centered counseling with YSUs at e-mental health services [29,41,66,67]. *Service-modality factors increasing effectiveness* were consistent with the views of other e-mental health professionals and their belief that text-based interventions are less appropriate for primary intervention of complex mental health issues and, instead, better positioned as adjunctive interventions that support self-management and existing face-to-face work [49,50,68,69]. Participants’ views also confirmed well-established notions about the advantages of text-based communication for users in processing, planning, and reviewing messages before sending them [61] as well as research indicating the superiority of having multiple sessions of TBOC rather than single sessions [54]. Counterintuitively, however, participants reported that TBOC services were effective in *crisis* situations. One possibility is that the participants considered effectiveness from the perspective that some crisis presentations may benefit from fast support irrespective of modality-related issues (eg, child abuse risk).

Participants’ views confirmed *factors perceived to decrease effectiveness* as a domain describing negative predictors of TBOC outcome. One theme, *interpersonal factors decreasing effectiveness*, partly described challenges in the online counselor’s management of clinical activities in a TBOC environment, which has some support in the literature. For example, participants’ views about the occurrence of goal setting issues are consistent with evidence that online counselors often underutilize goal exploration processes [41,52,55], whereas *misunderstandings* commonly stem from a *lack of nonverbal conversational cues* on text-based modalities [25,32,40,41,45,46]. Conversely, some participants’ perspectives were unique to the literature. For example, *counselors going off topic or being unsure how to help* is a plausible consequence in light of the commonality of working with complex presentations and goal setting issues on these modalities [49-53]. Similarly, the *poor conversion of counseling into postsession action* may be related to YSU motivation or online counselors struggling to bridge sessions together. Interestingly, the view that short-term outcomes decrease TBOC effectiveness contradicts evidence of the long-term outcomes possible on these modalities [33,60-62]. One possibility is that short-term outcomes were conceptualized as being undesirable when working with more complex presenting problems.

Participants also believed that interpersonal challenges affecting YSUs may reduce TBOC effectiveness, which were unique findings of the study. For example, participants suggested that *rupture in rapport* affected a minority of YSUs with more complex presentations featuring attachment trauma when service boundaries were altered or enforced, which is credible, given the safety-related reasons they select TBOC services. Likewise, a YSUs’ *low readiness, motivation, and self-confidence* to take action toward change might be expected to occur more frequently, given the help-seeking reluctance commonly observed among young people [23,29,31,66,67].

The second theme, *service-modality factors decreasing effectiveness*, described technical and environmental challenges to service efficiency. Herein, *working with more complex problems* was perceived to reduce TBOC effectiveness, consistent with the views of other e-mental health professionals in the literature [49,50]. This is curious, given evidence that these interventions can produce positive long-term outcomes for depression and anxiety presentations [42,68-70]. One possibility is that modality-related limitations become more difficult to manage with increased problem complexity. Indeed, in their study, Dowling and Rickwood [50] found that online counselors had a preference for nondirective person-centered interventions over directive interventions, such as cognitive behavioral therapy, because of time restrictions created by the slow pace of text-based communication and YSUs multitasking while using TBOC services. Correspondingly, participants reported a quadriad of themes that complicated intervention with complex presenting problems: (1) *challenges assessing presentations* that arise from TBOC services having *too much anonymity or few barriers*, (2) the *slow pace or lack of time to make a difference* when using text-based communication, (3) the *lack of nonverbal conversational cues* that can create *misunderstandings* in text-based communication, and (4) *environmental distractions* including *technical or connectivity issues* that affect a YSU’s focus and attention on a session and its impact on session time. These modality-related limitations mirror those reported by other e-mental health professionals in the literature [41,49,50,52,55,56]. In addition, participants held a unique view that TBOC services have *too much anonymity or few barriers*, suggesting a paradoxical interplay between reducing help-seeking barriers and reduced counseling effectiveness. Finally, participants viewed *poor timeliness of response* to service user requests to reduce TBOC effectiveness, which appears to be a common experience and complain by YSUs who use TBOC services [26].

### Research Implications for Online Counselors

Our findings have several implications for online counselors working with YSUs accessing TBOC services. Knowledge of *user characteristics* and *selection factors* may provide insight about possible clinical targets for counseling work, especially when a YSU’s presenting reasons are unclear. For example, *user characteristics* may provide clues to help with early assessment and intervention targets. Furthermore, online counselors may benefit from training in interventions for common *mental health* *syndromes* and *perceived social difficulties* that also consider a YSU’s *safety* and *avoidance motivation*. Similarly, knowledge of the *factors perceived to increasing and decreasing effectiveness* may provide insight about the fit between presenting problems and the modality-related limitations of TBOC services. For example, online counselors may experience better *fit* when presenting problems are lower in complexity and require less assessment, expectations and goals are actively negotiated, and users are encouraged to access over time to allow time to balance general counseling and the provision of self-management strategies. In contrast, online counselors may struggle against the quadriad of modality-limitations reported on TBOC services when more complex presenting problems are encountered. In these cases, using precounseling assessment questionnaires may allow online counselors to compensate for some modality-limitations to allow for a better use of session time and graduation to live counseling modalities or more specialized services. Finally, exploring methodologies to reduce *poor timeliness of response* to YSU requests (eg, feedback about delays, presession activities while waiting, and support alternatives), while providing them with feedback about the strengths and limitations of TBOC modalities, may help both parties to work together in harmony to improve outcome.

### Research Limitations

There are 3 main limitations in this study that need to be considered in relation to its findings. First, the use of mini focus groups may have produced fewer themes and less diversity in participant views than if using traditionally sized focus groups. Second, the semistructured nature of the interview may have resulted in missed opportunities to develop greater depth and stronger thematic narratives, compared with using a less structured interview format. Future research should consider using broader questions to overcome this limitation. Finally, although this study was designed to be a pilot preceding a large-scale study, incorporating participants into the data refinement stage of analysis using a Delphi method would likely reduce interrater bias and increased the validity of findings and their interpretations [73].

### Conclusions

This study examined e-mental health professionals’ perspectives about the factors that motivate YSUs to select TBOC services and influence the effectiveness of these services. Participants believed that YSUs commonly select TBOC services to increase their sense of safety in response to concerns about their privacy, themselves, and the help-seeking process. Participants also believed that using TBOC services allowed YSUs to increase their social support and access to mental health services, with nil-to-high expectations of the outcome. Factors perceived to increase the effectiveness of TBOC services were the online counselor’s interpersonal skills, leveraging the information processing benefits of text-based communication, and working with less complex presenting problems or referring YSUs with more complex issues to services better capable of supporting their needs. Factors perceived to decrease the effectiveness of TBOC services were interpersonal issues related to goal setting, postcounseling direction, and ruptured rapport. Service-modality factors decreasing effectiveness were ineffective technique, working with more complex presenting problems, poor timeliness in responding to YSU requests, and various limitations related to counseling in a text-based environment. Online counselors would profit from being aware of these factors to provide YSUs with better education, assessment, and interventions on TBOC modalities.

## Appendix

Multimedia Appendix 1 Pilot study semi-structured interview questions.

Multimedia Appendix 2 Consolidated criteria for Reporting Qualitative research (COREQ) checklist.

Multimedia Appendix 3 Overview of themes identified by text-based online counseling providers regarding young service users’ selection factors.

Multimedia Appendix 4 Overview of themes related to the factors perceived to increase effectiveness that were confirmed and identified in the study.

Multimedia Appendix 5 Overview of themes related to the factors perceived to decrease effectiveness domain that were confirmed and identified in the study.

## Abbreviations

e-mental: electronic mental
TBOC: text-based online counseling
YSU: young service user
