# Supplementary material for: Exploring Mental Health Professionals’ Perspectives of Text-Based Online Counseling Effectiveness With Young People: Mixed Methods Pilot Study
Source: JMIR Ment Health. 2020 Jan 29;7(1):e15564. doi: 10.2196/15564 (PMC7016626; doi:10.2196/15564)
Supplement: Multimedia Appendix 1 [file mental_v7i1e15564_app1.docx]

**Expert Panel Semi-Structured Interview**

INTERVIEW INTRODUCTION:

Welcome and thank you for taking part in this research study. In this interview, we are asking for your expert opinion about the psychotherapeutic mechanisms involved in a young person's use of Text-Based Online Counselling Services (TBOCS)(e.g., email, webchat, live forum). Your answers will contribute to a larger body of research we are conducting about why young people **select**, **use**, and potentially **stop** **using** TBOCS, especially when presenting with complex problems that may be less serviceable with the limitations of these modalities.

DOMAIN 1: Selection Factors

There is a lot of research about the factors that make TBOCS an attractive help-seeking option for young people. From our clinical experience and literature research, these can be clustered into three broad themes of belief that underpin initial contact behaviour with such services: ***Accommodation beliefs; Safety and Avoidance beliefs,*** *and****; Efficacy beliefs****.*

ACCOMMODATION BELIEFS:

***Accommodation beliefs*** are beliefs about how easy TBOCS' are to incorporate into various facets of one's life, compared to face-to-face services. Some major *Accommodation beliefs* appear to be:

- **Cost** (i.e. how affordable TBOCS are);
- **Accessibility** (i.e. how easy counselling services are to access), and;
- **Flexibility** (i.e. ease of fitting appointments around one's responsibilities and lifestyle).

QUESTIONS

1. How does this fit with your understanding and experience? Are there any other major factors in this cluster you're aware of or have encountered?
2. In a clinical setting, what would you expect to see or hear a young person say or do, to know that you've encountered an *Accommodation belief*?

SAFETY AND AVOIDANCE BELIEFS:

***Safety and Avoidance beliefs*** are those about TBOCS' feeling more approachable than face-to-face services. Some major *Safety and Avoidance beliefs* appear to be:

- **Privacy** (i.e. one's information will be kept confidential within TBOCS and/or one will be less likely to be seen to attend services in the community);
- **Anonymity and Invisibility** (i.e. one will be able to contact TBOCS' with minimal identifying information that may lead to being identified in the community);
- **Fast Distress Response** (i.e. one can contact at the moment of heightened distress);
- **Control** (i.e. feeling more in control of the interaction and when they can leave);
- **Interpersonal** **or Symptom** **avoidance** (i.e. one will experience less discomfort than talking to an in-person clinician or from interacting due to perceived social deficits), or;
- **Service** **avoidance** (i.e. one perceives TBOCS as a more responsive/more efficacious/ less uncomfortable/‘last resort’ option than existing or past service experiences).

QUESTIONS

1. How does this fit with your understanding and experience? Are there any other major factors in this cluster you're aware of or have encountered?
2. In a clinical setting, what would you expect to see or hear a young person say or do, to know that you've encountered a *Safety and Avoidance belief*?

EFFICACY BELIEFS:

***Efficacy beliefs*** are those that pertain to beliefs about how effective TBOCS' are as a clinical intervention. Some major *Efficacy* *beliefs* appear to be:

- **Short-term Retrospective efficacy** (i.e. TBOCS has helped in the past with short-term problems);
- **Long-term Retrospective efficacy** (i.e. TBOCS has helped in the past with a long-term problem);
- **Generalised efficacy** (i.e. TBOCS use is believed to function similarly to other therapeutic services irrespective of service type, delivery modality, or prior contact experience);

QUESTIONS

1. How does this fit with your understanding and experience? Are there any other major factors in this cluster you're aware of or have encountered?
2. In a clinical setting, what would you expect to see or hear a young person say or do, to know that you've encountered an *Efficacy belief*?

DOMAIN REVIEW

1. Would you agree that these three broad beliefs underpin initial contact behaviour with a TBOCS? Would you add any other variables?

______________________________________________________________________________

DOMAIN 2: Efficacy Factors

While there is research suggesting that e-Mental Health Services were initially established for reasons related to service accessibility and low help-seeking attitudes, it is our belief that a high proportion of presentations on TBOCS may also be associated with ***Avoidance***-based presentations that stem from ***Safety and Avoidance beliefs***. If true, we theorise that young people experience a *reinforcement of help-seeking behaviour* on a TBOCS as a result of relief from some anxiety and avoidance of factors underpinning this presentation.

QUESTIONS

1. How does this fit with your understanding and experience?
2. In a clinical setting, what would you expect to see or hear a young person say or do, to know that you've encountered an **Avoidance**-facilitated presentation?

Where ***Avoidance*** plays a role in initiating contact with a TBOCS, research has predominately suggested that contact with a TBOCS can be traced to **Engagement** **Variables**, like positive therapeutic alliance and natural counselling benefits (e.g. feeling validated, normalised, catharsis). As such, we theorise that young people experience a *reinforcement of* *help-seeking behaviour* on a TBOCS as a result of these factors.

QUESTIONS

1. How does this fit with your understanding and experience?
2. In a clinical setting, what would you expect to see or hear a young person say or do, to know that you've encountered an **Engagement** **Variable**?

For more complex mental health presentations related to ***Avoidance***, we theorise that longer-term TBOCS use may have negative therapeutic effects related to the limitations of TBOCS and lack of confrontation of avoidance variables. This ***Plataeu Effect*** is believed to result from various poor goodness-of-fit factors (e.g. low help-seeking clients not ready for work, poor modality-problem fit, iatrogenic effects, reduced efficacy of **Engagement Variables** over time) that challenge a user's ***Efficacy Beliefs***.

QUESTIONS

1. How does this fit with your understanding and experience?
2. In a clinical setting, what would you expect to see or hear a young person say or do, to know that you've encountered a ***Decay Effect***?

Some research suggests that a number of TBOCS are used in an ongoing way by young people with complex mental health issues and urgent presentations. Given some of the limitations of TBOCS in terms of helping with the complexity of such presentations over time, we theorise that many people who engage in an ongoing way despite a lack of progress, may experience a **Reprioritisation** **of the Therapy Relationship over Goals**. That is, **Engagement** **Variables** become a greater focus than original therapy goals, which shift in importance. This is also theorised to reinforce ***Avoidance*** variables.

QUESTIONS

1. How does this fit with your understanding and experience?
2. In a clinical setting, what would you expect to see or hear a young person say or do, to know that you've encountered a **Reprioritisation** **of Therapy Relationship over Goals** presentation?

DOMAIN REVIEW

1. Are there any other observations or comments you’d like to share related to your professional experience in this area, that we may have not considered?

*Thank you for your time and assistance today.*
