# Supplementary material for: Exploring Mental Health Professionals’ Perspectives of Text-Based Online Counseling Effectiveness With Young People: Mixed Methods Pilot Study
Source: JMIR Ment Health. 2020 Jan 29;7(1):e15564. doi: 10.2196/15564 (PMC7016626; doi:10.2196/15564)
Supplement: Multimedia Appendix 3 [file mental_v7i1e15564_app3.docx]

Overview of themes identified by text-based online counseling providers regarding young service users’ *selection factors*.

| Domains/themes | | | Strength of theme | Examples of theme |
| --- | --- | --- | --- | --- |
| **Selection factors** | | | | |
|  | **Safety** | | **Very strong** |  |
|  |  | *Increased privacy* | Very strong | *Absolutely. Sitting in the waiting room at the psychologist's, in the eye street, so to speak, people know exactly why they’re there.* *So I think there's far more privacy and I think that's one of the advantages of [TBOC].* |
|  |  | *Increased control and autonomy* | Strong | *I think it relates to a lot of these things under the safety category that maybe just really identifying that this is a medium they feel control. If they want anonymity, they have the potential to have that. If they engage with other services, they might not get that.* |
|  |  | *Increased anonymity* | Strong | *What most of the kids have told us about anonymity is that they feel uncomfortable having to present themselves in person or talk to someone in person. They feel safer when people don't know who you are. If people don't know who you are they can't do anything to you. They at times make you do things you don't want to do, like medication or going to some kind of facility. It’s those kinds of things that kids have commonly told us.* |
|  | **Accessibility** | | **Very strong** |  |
|  |  | *Convenience and flexibility* | Very strong | *There's a number of studies now that say [YSUs’] generation don't care [who they see for professional support], it's the most convenient person I'll go to. If I'm working in the city, I will see the GP in the city. If I'm at home, I'll see my local one, but I don't care in terms of the relationship with the continuity of care, I'm used to these.* |
|  |  | *Faster access to counseling* | Very strong | *Some people can wait like six weeks, two months, three months to get an appointment. Whereas web, for example, you can just jump on and wait anywhere from two minutes through to an hour.* |
|  |  | *Affordability* | Strong | *I think cost is a major thing as well [for YSUs]. I don't think a lot of the clientele would actually pay the cost themselves, hence if they had to attend a traditional service, they would need to engage their parents or their carer to actually do that. This allows them to be independent in actually entering the service.* |
|  |  | *Sourcing one’s own support* | Strong | *I probably see entry into the system and probably that using [TBOC] is the least [effort]... To get on face-to-face here, you have to actually engage, make an appointment, and potentially even go to a GP or someone else to be referred to the service. However, you've got absolutely no barriers to an actual entry point into a service using [TBOC].* |
|  |  | *Circumnavigating waiting lists* | Strong | *I get feedback from [YSUs] that it's easier to engage with [TBOC], even if I'm encouraging them to continue developing their relationship with face-to-face providers, given that can be difficult sometimes making appointment and organizing that in advance since they can book up quite quickly.* |
|  |  | *Access to counseling in areas of low service density* | Moderate | *These services are obviously a lot more accessible to people in rural /remote areas.* |
|  |  | *Preferred way to communicate* | Weak | *A young person just prefers to engage with technology and to engage in an online world, as opposed to telephone or face-to-face. With that, the motivation for choosing and may purely come from that is just the known space where they already live a lot of their life. It is to seek help in the online world, either to some mere extensional for that. That familiarity and preference.* |
|  |  | *Overcoming transportation issues* | Weak | *[YSUs have] to travel sometimes long distances, so that clearly is an issue. But also, you know, transport can be an issue even within the capital city. It’s difficult for young people who don’t have a car to get around.* |
|  | **Avoidance motivation** | | **Very strong** |  |
|  |  | *Being overheard or seen attending a service* | Very strong | *Specifically, I was thinking about a young person who was contacting on [TBOC] because she didn't want to go to the school counsellor because of the perception of what all the kids would say, or think, or do. Because that room is public in their school and you can see who's going in there. In terms of privacy, I guess that limits that. Nobody sees what's happening. I guess that would be stigma as well.* |
|  |  | *Minimizing intense or difficult emotions* | Strong | *For some young people, they've certainly chosen [TBOC] first because they feel some shame or embarrassment by talking it over the phone or in person.* |
|  |  | *Privacy and security concerns* | Strong | *I think [YSUs] still do have some concerns about privacy online as well because that's certainly been asked and questions like that, “Who else can see this?” “Is this information stored?”* |
|  |  | *Counselor reaction* | Strong | *[Many YSUs fear the idea that] “if I talk to somebody they'll come and take me to the nuthouse” and the consequence.* |
|  |  | *Challenging conversations* | Strong | *Depending on the particular topic or theme, it is something that carries its own perceived social stigma or if there is something that they've experienced they’ve not being supported with before, they might choose [TBOC instead].* |
|  |  | *Social interaction* | Moderate | *If you ask [a YSU], if you've spoken to a counsellor in person before they say, ‘no’. I feel they will tell you that … they prefer using web chat and they prefer to type it rather than say it out loud.* |
|  | **Expectations** | | **Strong** |  |
|  |  | *Few expectations/ referral* | Strong | *I think some [YSUs] may take the view that, well, “I just need something right now without really reflecting too much on the question of whether it was likely to be useful.” It's more of a need...I think often kids don't really focus that much on issues of efficacy and outcome. They tend to more focus on need in the moment.* |
|  |  | *High treatment expectations* | Moderate | *In thinking of the emails or people that come on to our service who are engaged with the number of services, [I’ve heard comments like], “Yes, I've tried everything and nothing's worked.” So whether that they are seeing this is a last resort and for whatever reasons that they feel that they are not getting the help that they need through the other avenues, they come to online services.* |
|  |  | *Prior successful interaction* | Moderate | *One example that comes to mind is on email [counselling]. When a [YSU] who may have had a short-term engagement via email [counselling] previously maybe a number of years ago, comes back and says, “I was in contact with you guys a while ago. I've got another problem,” indicating that yet they do believe that there was some short-term efficacy around that issue. They've come back again for your help around a specific issue.* |
|  |  | *Equivalent to other counseling* | Moderate | *There's a possibility some young people actually feel like [TBOC is] more effective than other services out there.* |
