# Supplementary material for: Exploring Mental Health Professionals’ Perspectives of Text-Based Online Counseling Effectiveness With Young People: Mixed Methods Pilot Study
Source: JMIR Ment Health. 2020 Jan 29;7(1):e15564. doi: 10.2196/15564 (PMC7016626; doi:10.2196/15564)
Supplement: Multimedia Appendix 4 [file mental_v7i1e15564_app4.docx]

Overview of themes related to the *factors perceived to increase effectiveness* that were confirmed and identified in the study.

| Domains/themes | | | Strength of theme | Examples of theme |
| --- | --- | --- | --- | --- |
| **Factors increasing effectiveness** | | | | |
|  | **General therapeutic benefits** | | **Very strong** |  |
|  |  | *Feeling listened to and understood* | Moderate | *People [using TBOC] do get a sense that the counsellor is there to help them, and they're sympathetic, and is listening.* |
|  |  | *Catharsis or debriefing* | Weak | *There's potentially another [benefit] in terms of just debriefing if you potentially say a bullying incident or something and you just want to talk to somebody and [TBOC] is the way to do it. You don't particularly or may not actually be seeking active treatment or you just need to debrief.* |
|  |  | *Feeling normalized and validated* | Weak | *What happens when you first approach [a TBOC] service is tremendously important. That initial exchange—whether it is something which... makes you feel comfortable by the way in which it is done is really quite critical. So it's not just respect, it's experience of warmth of acceptance in an interaction that make you feel safe.* |
|  | **Positive service-modality factors** | | **Very strong** |  |
|  |  | *Working with less complex presenting problems* | Strong | *I believe that text-based online counselling may not benefit some clients with complex mental health issues, developmental delays and/or learning difficulties.* |
|  |  | *Stepping stone to more intensive counseling support* | Strong | *I think that sometimes there are some young people, maybe not a lot, but there are some young people for who these types of modalities can be a really important stepping stone into other supports and areas. That if they had never used those modalities, and that type of help seeking, maybe they never would have got to face to face counselling. I think it has got a really important role that can be with young people with significant mental health concerns as well.* |
|  |  | *Ease of expression or thought organization* | Moderate | *[YSUs] will tell you that they prefer using web chat and they prefer to type it rather than say it out loud. Again, I think that for some kids it's a processing thing as well it gives them—I've had that feedback from some as well. I can actually think about what I want to say.* |
|  |  | *Crisis use* | Moderate | *With the idea around so many [YSUs] contacting on web for self-harm and suicide, and even over email, that seems to be such a big theme that they are able to get a quicker response.* |
|  |  | *Written record of information* | Moderate | *Sometimes, I get feedback at the end of the session that [YSUs] are going to copy and paste certain parts of the transcript so that they can remember it or just use some of the strategies we talked about in the future.* |
|  |  | *Problem self-management strategies* | Weak | *I see these services and services that we give... as... supporting self-management or self-control and I think that really comes across... But some other mental health services that I might have come across may not necessarily have quite that perspective.* |
|  |  | *Therapist assistance* | Weak | *Some people just don't like dealing indirectly, like any kind of automated [*e-mental health*] service. [They] want to talk to somebody and get a solution to that problem in person.* |
|  | **Persisting with counseling to increase benefit** | | **Moderate** | *With any web-based intervention, a key element is the extent that the person persists in engaging with it.* *If someone is depressed, they may be sceptical that anything will help, and will be turned off by even a minor perceived issue with a response by a counsellor... a sense that the person has not understood or that the response is unhelpful.* |
