# Supplementary material for: Exploring Mental Health Professionals’ Perspectives of Text-Based Online Counseling Effectiveness With Young People: Mixed Methods Pilot Study
Source: JMIR Ment Health. 2020 Jan 29;7(1):e15564. doi: 10.2196/15564 (PMC7016626; doi:10.2196/15564)
Supplement: Multimedia Appendix 5 [file mental_v7i1e15564_app5.docx]

Overview of themes related to the *factors perceived to decrease effectiveness* domain that were confirmed and identified in the study.

| Domains/themes | | | Strength of theme | Examples of theme |
| --- | --- | --- | --- | --- |
| **Factors perceived to decrease effectiveness** | | | | |
|  | **Interpersonal factors reducing effectiveness** | | **Very strong** |  |
|  |  | *Problem not improving or ineffective techniques* | Strong | *If all [YSUs] are experiencing is analogous to sitting down with someone having a nice cup of tea, then they won't actually see long term gains from it. And in fact you can develop a dependence on that is all of the services find that they have some people who really heavy users and who appear to just like the sessions, but they're not actually changing their lives at all. It's a bit of a cycle.* |
|  |  | *Rupture in rapport* | Strong | *I think requesting a new counsellor to work with, or adding a second counsellor, is really big [on TBOC].* |
|  |  | *Focus on rapport instead of goals* | Strong | *Yes, I think that can happen with longer-term users they can develop a rather dependent relationship with particular counsellors... when I was involved with [a TBOC service], young people were allowed to ask for and re-engage with particular counsellors, and there was some evidence that at least some young people had become attached to and dependent on particular counsellors. I'd say it's quite a small minority but because they tended to be frequent users, that could become well known.* |
|  |  | *Poor conversion of counseling into postsession action* | Strong | *We know that therapist-assisted web-based interventions are as good, well-established now... Where you get a little bit of lower impact with the web interventions for adults is where they uncoached and they have a fidelity issue. But it's a really interesting fidelity issue. It's not a fidelity in what's being given to them, it's a fidelity in what they expose themselves to... [it is about] their willingness to actually convert [the session content] into some sort of action.* |
|  |  | *Desiring more service control* | Strong | *Current policy at the service dictates a maximum time allowed for email counselling before encouraging the [YSU] to move to phone or web [counselling]. The prospect of just having a limited number of emails may be off putting.* |
|  |  | *Incongruent client-counselor goals* | Moderate | *A [YSU] might perceive the response from the service as being unhelpful to them. For example, being continually encouraged to contact by phone. Perhaps this is frustrating to a young person and they are not given the opportunity to continue to engage the way they want to on [TBOC]... Another possibility is that the young person is not finding it helpful and is unwilling to contact via another medium or seek face to face counselling as they are not feeling comfortable or ready to do this.* |
|  |  | *Unclear goals or reasons for counseling* | Moderate | *There’s those [YSUs] that do connect with the service around a specific issue and may do so daily, or multiple times in a couple of weeks, around an issue and engaging with a number of different counsellors... And they feel good. It feels great to be heard. But the resolution of the issue, if there is an underlying issue, may not come quick enough for them because of a lack of structured purpose for therapy.* |
|  |  | *Misunderstandings or literacy issues* | Moderate | *The [YSU] may not have effective text-based online communication skills and/or comprehension, leading to limited engagement in online sessions- this could potentially lead to frustration from the young person and disengaging from the service.* |
|  |  | *Low readiness, motivation, and self-confidence* | Moderate | *Perhaps like all counselling mediums the young person may not be ready for counselling or change.* |
|  |  | *Counselor going off-topic or being unsure how to help* | Moderate | *[Another factor reducing effectiveness on TBOC is] perhaps, a lack of clarity on the counsellor’s behalf about how they can work [with a YSU] within a particular framework of practice, depending on the particular service that you're working in.* |
|  |  | *Outcomes with only short-term benefit* | Weak | *[Another factor reducing effectiveness on TBOC is] crisis only work, with no counselling in between, so it’s just meeting that initial need without any long-term benefit.* |
|  | **Negative service-modality factors** | | **Very strong** |  |
|  |  | *Working with more complex problems* | Very strong | *I think that [one] of the reasons a young person may not benefit from [TBOC is a] lack of fit between problem and modality... it feels like some issues, particularly complex mental health concerns, are too complex to adequately address by [TBOC].* |
|  |  | *Poor timeliness of response* | Strong | *I think the other thing too that you might like the [YSU] to see is how quick they actually get a response as well. When I say that, it's time to first response and time to actual treatment... One of the papers I published during my PhD actually looked at response times to email [YSUs] and it found a significant difference in [YSUs] that reused the service... The 36 hour mark was the point where we found that the response was actually statistically significant.* |
|  |  | *Challenges assessing presentations* | Strong | *I think therapists feel very uneasy about [not having assessment data], because they bear a lot of professional responsibility. It's difficult to manage that responsibility when you don't have all the data that you want.* |
|  |  | *Too much anonymity or few barriers* | Strong | *Sometimes reducing the barriers too much is a bad thing because you end up with some unnecessary or flippant approaches to services and that, by reducing all barriers to service. And we see that rationing is an important part of health service, there's no doubt about it, even though we don't want to talk about it. The concept of putting in a GP co-payment, that was a form of rationing, putting some sort of barrier there. You're making sure that if you wanted to access the service, that you had some really legitimate reason and that by removing all barriers you take that away to a certain degree.* |
|  |  | *Lack of nonverbal conversational cues* | Moderate | *It can be difficult to support the [YSU] if they are disassociating and not providing written feedback to the counsellor about what is happening where as visual and audio cues could assist a counsellor in understanding what is happening for the [YSU] in another type of counselling setting.* |
|  |  | *Misunderstandings or literacy issues* | Moderate | *The [YSU] may not have effective text-based online communication skills and/or comprehension, leading to limited engagement in online sessions- this could potentially lead to frustration from the young person and disengaging from the service.* |
|  |  | *Environmental distractions* | Moderate | *[YSUs] may have limited access to the technology required and therefore can only access at specific times. For example, late at night or early in the morning when family is asleep when they are tired, and therefore not fully able to engage in counselling. Or during school or class times in which sessions may get cut short when class is over or a teacher catches them. Or evenings when they are supposed to be doing homework and are potentially multi-tasking and getting distracted.* |
|  |  | *Slow pace or lack of time to make a difference* | Moderate | *I don't believe text based services can be as effective as phone or face to face services for [YSUs] needing support with grounding exercises for panic attacks or flashbacks given the slower pace of web and other challenges with facilitating these exercises online.* |
|  |  | *Technical or connectivity issues* | Weak | *Lag and poor internet connection can make the process frustrating for [YSUs].* |
